# Correction to “Adult and pediatric relapsing multiple sclerosis phase II and phase III trial design and their primary endpoints: A systematic review”

**DOI:** 10.1111/cts.70052

**Published:** 2024-10-21

Katsutoshi Hiramatsu, Hideki Maeda. Adult and pediatric relapsing multiple sclerosis phase II and phase III trial design and their primary end points: A systematic review. *Clin Transl Sci*. 2024;17:e13794.

In Figure 2 of the text “Records excluded (n = 232) with reasons:” was incorrect. This should have read: “Records excluded (n = 260) with reasons:”

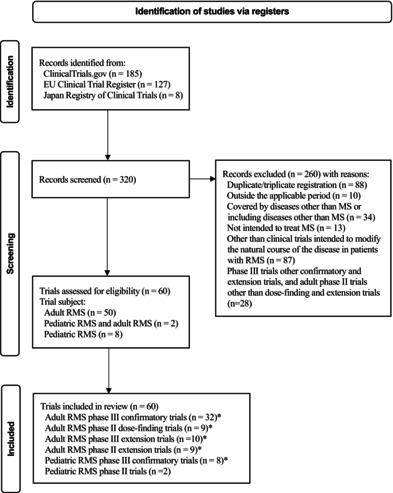

We apologize for this error.

